# The Structure, Evolution, and Expression Patterns Analysis Reveals the bHLH Members Associated with Powdery Mildew Resistance in Rubber Tree

**DOI:** 10.3390/plants14213244

**Published:** 2025-10-22

**Authors:** Xiaokang Fan, Xiaoling Tang, Yiying Lu, Yan Zhang, Cuicui Wang, Yu Zhang, Lifeng Wang

**Affiliations:** 1 College of Tropical Agriculture and Forestry, Hainan University, Danzhou 571737, China; fanxiaokang2023@163.com (X.F.); tangxlrri@163.com (X.T.);; 2 Sanya Institute of Breeding and Multiplication, Hainan University, Sanya 572024, China; 3 Key Laboratory of Biology and Genetic Resources of Rubber Tree, Ministry of Agriculture and Rural Affairs/State Key Laboratory Incubation Base for Cultivation & Physiology of Tropical Crops/Rubber Research Institute, Chinese Academy of Tropical Agricultural Sciences, Haikou 571101, China

**Keywords:** *Hevea brasiliensis*, bHLH, evolution patterns, expression pattern, powdery mildew

## Abstract

The basic helix–loop–helix (bHLH) transcription factors play a crucial role in plant development and stress resistance. Elucidating the structure and function of bHLH family members related to rubber tree powdery mildew (*Erysiphe quercicola*) is essential for breeding disease-resistant rubber tree varieties. In the rubber tree (*Hevea brasiliensis* Muell. Arg.) variety CATAS73397, 204 HbbHLH transcription factors were systematically identified at the genome level and classified into 15 subfamilies through evolutionary analysis. The expansion of this family was primarily driven by whole-genome duplication (WGD). Based on RNA-seq data from leaves infected with powdery mildew, 11 *HbbHLH* genes responsive to infection were identified. Phylogenetic analysis examined the evolutionary relationships between rubber tree *bHLH* genes and disease-resistant *bHLH* genes from other plants. Promoter analysis of the 11 differentially expressed genes revealed abundant cis-elements associated with light responses, hormones, and transcription factor binding. Quantitative Real-time polymerase chain reaction validation indicated that *HbbHLH87* and *HbbHLH162-2* were significantly downregulated during infection, whereas *HbbHLH25* was significantly upregulated. These three genes exhibited strong responses to methyl jasmonate (MeJA) and salicylic acid (SA) treatments, suggesting their involvement in jasmonic acid and SA signal transduction pathways during the immune response. This study provides important insights into the molecular mechanisms underlying disease resistance in rubber trees and identifies potential targets for breeding disease-resistant varieties.

## 1. Introduction

The rubber tree powdery mildew (*Erysiphe quercicola*) has a severely adverse impact on the growth of rubber trees (*Hevea brasiliensis* Muell. Arg.). Powdery mildew is one of the primary leaf diseases affecting rubber trees [1]. Since its initial discovery in Java, Indonesia, in 1918, it has spread throughout various rubber-growing regions in Asia and Africa [2]. Currently, there is a lack of disease-resistant varieties. Under favorable natural conditions, such as specific weather and seasonal factors, large-scale epidemics occur, resulting in significant reductions in rubber production. For example, a large-scale outbreak of powdery mildew on rubber trees in Xishuangbanna, Yunnan Province, led to a 20% decrease in natural rubber output [3]. Powdery mildew disease in rubber trees is characterized by rapid spread and a short infection duration [4]. The disease has been reported in various countries; notably, its outbreak in the Philippines was more severe than that of other diseases [5]. Presently, in addition to traditional chemical control, disease-resistant crop breeding, and the exploration of disease-resistant genes, new technologies such as disease outbreak prediction, remote sensing, and agricultural protection drones are also important tools for controlling powdery mildew outbreaks. The identification of disease-resistant genes for rubber tree powdery mildew is of great significance for preventing future outbreaks.

In recent years, research on resistance genes against rubber tree powdery mildew has yielded promising initial results. RNA sequencing analysis of leaves infected by powdery mildew fungi indicates that the Mildew resistance locus O (*Mlo*) gene is associated with susceptibility to powdery mildew disease [6]. HbMlo12 is a susceptible gene for rubber tree powdery mildew. It is regulated under the infection of the fungal pathogen causing rubber tree powdery mildew [7,8,9]. HbLFG1 (Lifeguard) promotes powdery mildew infection by suppressing plant immunity [10]. HbSGT1 (suppressor of G2 allele of skp1) and HbHSP90.1 (heat shock protein 90.1) form a complex that plays a crucial role in rubber tree resistance to powdery mildew, stress tolerance, and hormone signal transduction [11]. HbRPW8-a (Resistance to Powdery Mildew 8) was widely involved in the disease resistance process through the salicylic acid (SA) signaling pathway [12]. Certain initial achievements have made in the research study major disease-resistant disease-resistance genes; however, difficulties challenges remain rubber tree genetic breeding. Uncovering Identifying factors related to disease resistance and analyzing elucidating regulatory mechanism mechanisms underlying rubber tree powdery mildew resistance is crucial the effective and control of powdery this disease.

Among plants, basic helix–loop–helix (bHLH) transcription factors, the second largest family of transcription factors, play a crucial role in regulating plant growth and development, secondary metabolism, and adaptive responses to abiotic stresses. bHLH transcription factors consist of a basic DNA-binding region (b) and a helix–loop–helix region (HLH), and they possess a highly conserved domain containing approximately 50 to 60 amino acids [13]. Wei et al. identified 183, 231, and 571 bHLH transcription factors in the genomes of rice, corn, and wheat, respectively [14]. Wang et al. identified 180 members of the bHLH family in the rubber tree genome (not yet published) and presented the temporal and spatial expression patterns of the *HbbHLH* gene in 16 rubber tree varieties [15]. The role of bHLH transcription factors in disease resistance across various plants has been a research hotspot in recent years. Huang et al. identified CsbHLH085 as a resistance factor against citrus bacterial canker disease (*Xanthomonas citri subsp. citri* (*Xcc*)) [16]. Meng et al. discovered that OsbHLH6 regulates the SA and jasmonic acid (JA) signaling pathways to mediate rice resistance to rice blast (*Magnaporthe oryzae*) [17]. Yu et al. found that VabHLH137 promotes the biosynthesis of proanthocyanidin (PA) in grapes, significantly enhancing resistance to grape anthracnose (*Colletotrichum gloeosporioides*) [18]. Overexpression of *MdbHLH093* in tobacco leads to increased hydrogen peroxide accumulation and activation of the SA signaling pathway, thereby enhancing resistance to apple powdery mildew (*Podosphaera leucotricha*) [19]. The gene *CmbHLH87* was heterologously overexpressed in tobacco plants, which alleviated powdery mildew symptoms (*Podosphaera xanthii*) on tobacco leaves and simultaneously reduced bacterial concentrations at infection sites of bacterial pathogens such as bacterial wilt and bacterial blight (*Ralstonia solanacearum*) [20]. However, bHLH family members in rubber trees have not yet undergone systematic analysis, and *bHLH* genes related to resistance against diseases such as powdery mildew have not been screened and identified. Identifying bHLH transcription factors involved in disease resistance and analyzing their transcriptional regulatory mechanisms during rubber tree resistance to powdery mildew can provide a foundation for breeding resistant varieties and is of great significance for controlling powdery mildew in rubber trees.

This study presents a comprehensive identification and bioinformatics analysis of the bHLH family members in the rubber tree cultivar CATAS73397 genome at the genomic level. Additionally, transcriptional profiles from both field-grown rubber tree leaves infected with powdery mildew and those artificially inoculated were utilized to screen bHLH family members associated with powdery mildew resistance from multiple perspectives. A systematic analysis of the bHLH family in the rubber tree cultivar CATAS73397 genome was conducted, focusing on members directly related to disease resistance. This study also identified bHLH family members linked to rubber tree powdery mildew, establishing a theoretical foundation for subsequent functional validation. The screening and functional verification of bHLH transcription factors related to rubber tree powdery mildew will provide valuable theoretical guidance for the prevention and control of this disease.

## 2. Results

### 2.1. Identification and Structural Analysis of Members of the bHLH Family

**Figure 1:**
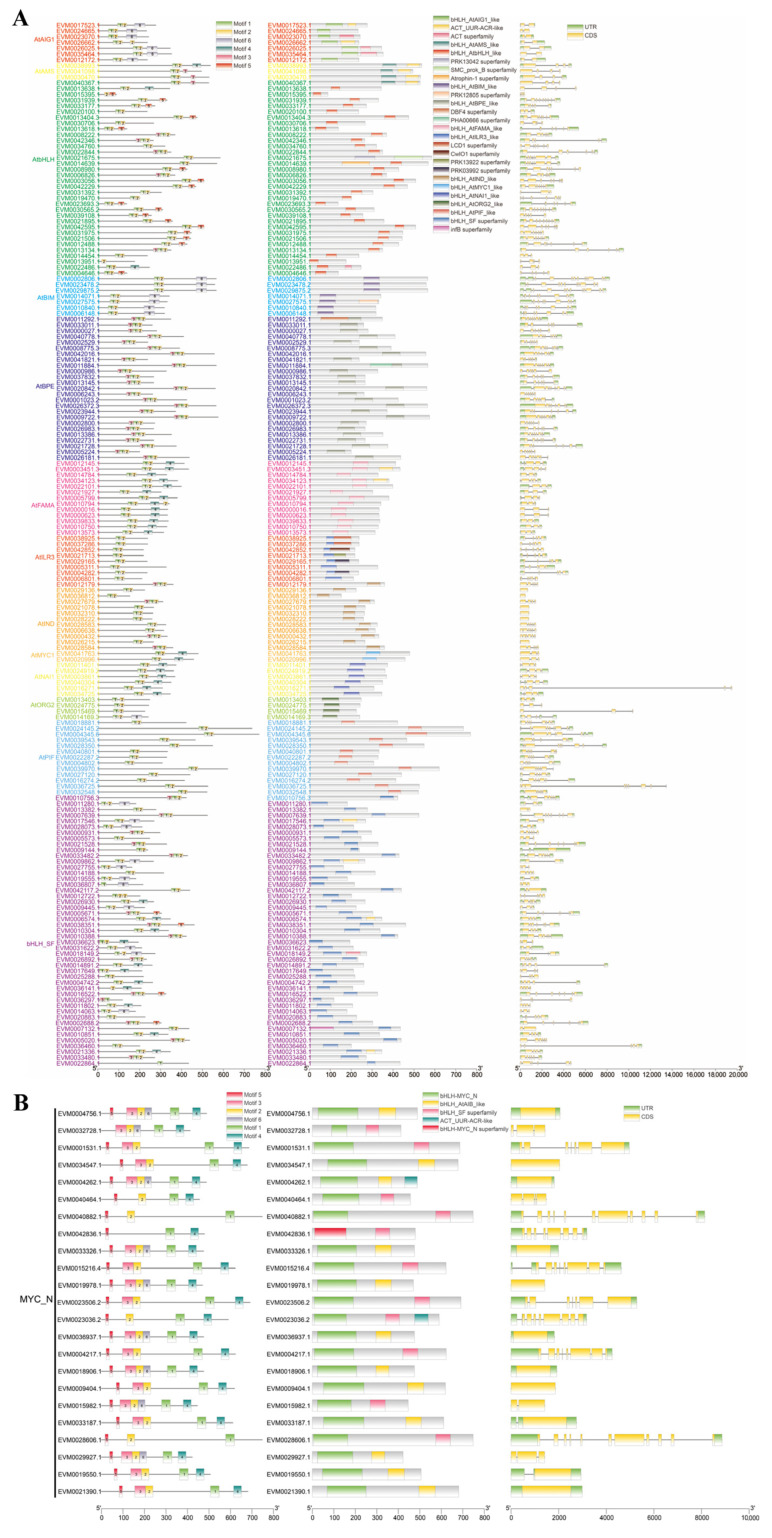
MEME and structure analysis of bHLH in *Hevea brasiliensis.* (**A**) A diagram of the single bHLH domain structure, MEME and sequence coding and non-coding regions were analyzed. (**B**) A diagram of the double bHLH domain structure, MEME and sequence coding and non-coding regions were analyzed.

A total of 204 bHLH family members were identified in the genome of the rubber tree cultivar CATAS73397. Among them, 181 members contained a single bHLH domain (Figure 1A), while 23 members possessed two bHLH domains (Figure 1B). Based on domain characteristics, the 181 single-domain bHLH members were classified into 13 groups (Figure 1A), whereas the 23 members with two conserved domains were grouped together and designated as MYC_N (Figure 1B). MEME motif clustering analysis revealed that motif arrangement patterns were similar within groups sharing the same domain type. For instance, the motif arrangement in the AtAIG1 and AtBIM groups was 1-2-6, whereas members of the AtbHLH group exhibited a motif pattern of 1-2-5. The number of exons among the 204 bHLH members ranged from 1 to 10. The physicochemical property results of 204 members of the bHLH family indicated that the range of the Instability Index was greater than 33.97–85.42, the range of the Aliphatic Index was 50.05–105.41, and the range of the Grand Average of Hydropathicity was −0.966 to −0.034 (Table S1). The subcellular prediction and localization analysis indicated that all bHLH members in this study were localized in the nucleus (Table S1).

### 2.2. Evolution and Collinearity Analysis of the bHLH Family

**Figure 2:**
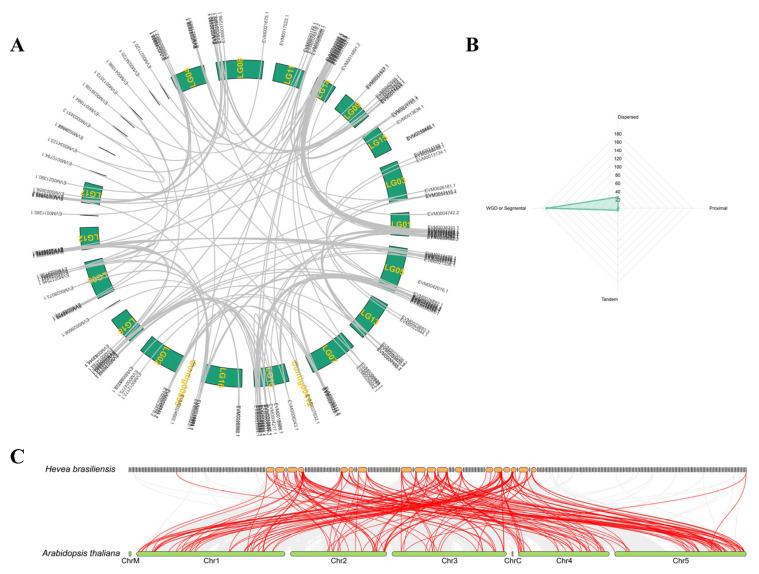
Synteny and duplication type analysis of HbbHLH family members in *Hevea brasiliensis*. (**A**) The arrangement and collinearity of bHLH on chromosomes. (**B**) Radar chart for statistics of duplication types of bHLH family members. (**C**) Collinearity analysis of bHLH sequences between *Hevea brasiliensis* and *Arabidopsis* genomes.

The replication types of the 204 members of the bHLH family were as follows: 171 bHLHs exhibited whole-genome duplication (WGD) or segmental duplication, 27 were dispersed, 5 were tandem duplicates, and 1 was proximal (Figure 2A). There was strong collinearity among the bHLH family members. Most collinearity genes were distributed across different chromosomes, consistent with the predominance of WGD or segmental duplication as the main replication types for bHLH family members (Figure 2B). The bHLH genes were widely distributed across multiple chromosomes. Based on these observations, it can be concluded that the expansion of the bHLH family was primarily driven by WGD or segmental duplication. Collinearity analysis between rubber tree and *Arabidopsis thaliana* revealed 121 rubber tree bHLH members and 99 *Arabidopsis thaliana* bHLH members that exhibited collinearity (Figure 2C).

### 2.3. Systematic Evolutionary Relationships Among Members of the bHLH Family

**Figure 3:**
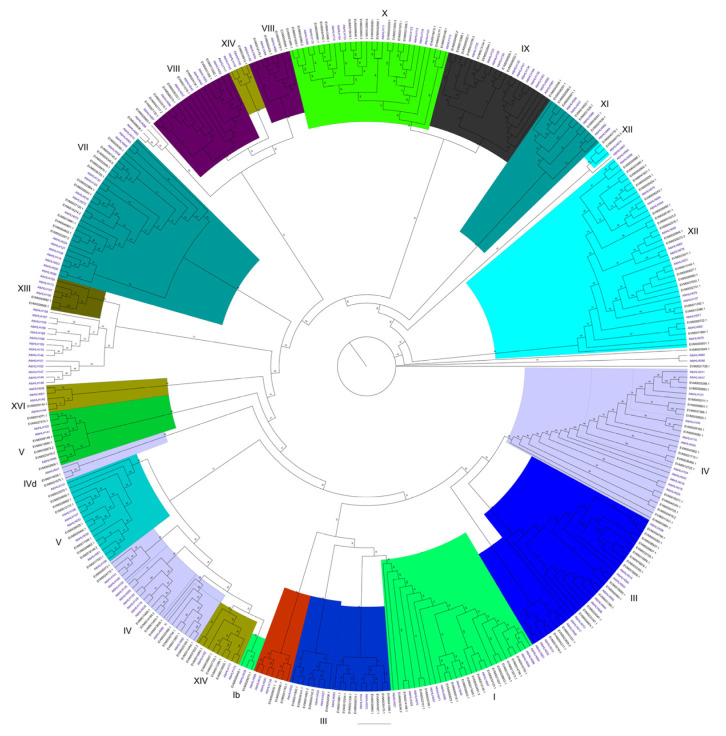
Phylogenetic analysis of the bHLH in *Hevea brasiliensis* and *Arabidopsis thaliana*.

To investigate the systematic evolutionary relationships among bHLH family members, an ML tree was constructed using the amino acid sequences of bHLH proteins from rubber trees and *Arabidopsis thaliana*. Based on the classification established for Arabidopsis thaliana, the 204 *bHLH* genes from the rubber tree were grouped into 21 clades, comprising a total of 15 subfamilies (Figure 3). Notably, two rubber tree bHLH members could not be assigned to any specific subfamily. The III, IV, V, XII, and XIV subfamilies each contained two distinct clades.

### 2.4. RNA Sequencing Analysis of bHLH Family Member Expression Patterns Following Powdery Mildew Treatment

**Figure 4:**
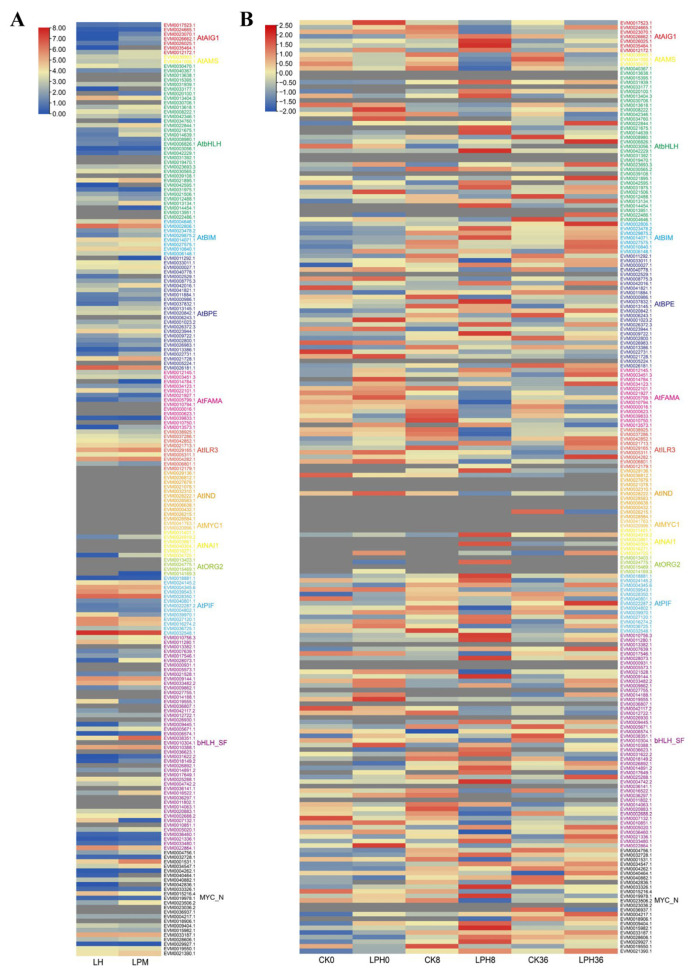
Expression patterns of *HbbHLH* in *Hevea brasiliensis* leaves under powdery mildew infection and healthy conditions. (**A**) Expression patterns of *bHLH* in infected and healthy leaves of powdery mildew disease. (**B**) Expression patterns of bHLHs in leaves following artificial inoculation with powdery mildew.

**Figure 5:**
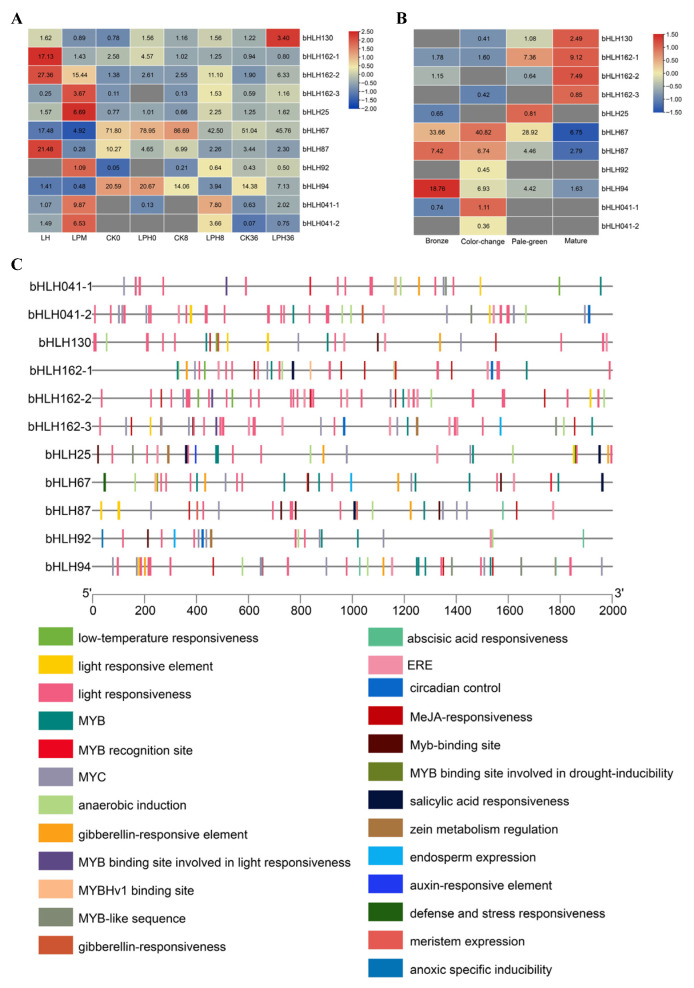
Expression patterns and cis-regulatory elements of bHLH transcription factors in powdery mildew response. (**A**) Heatmap of 11 differentially expressed *bHLH* genes in artificially inoculated and naturally infected samples. (**B**) Expression patterns of *bHLH* during different developmental stages of the leaf. (**C**) Distribution of cis-acting elements in promoter regions (2000 bp upstream of CDS).

The expression patterns of *bHLH* genes in rubber tree leaves infected with powdery mildew compared to uninfected leaves revealed that 51 *bHLH* genes exhibited significantly different expression levels, while 54 *bHLH* genes were not expressed in the leaves (Figure 4A). Among the *bHLH* gene family members, those containing the AtPIF-like domain tended to show higher expression in the leaves compared to other groups. RNA sequencing analysis of rubber tree leaves artificially inoculated with powdery mildew indicated that 50 *bHLH* genes were significantly differentially expressed, whereas 33 *bHLH* genes were not expressed at all in the leaves (Figure 4B). Furthermore, most bHLH family members with the AtIND domain were not expressed in the leaves. In contrast, most genes in the AtPIF group were highly expressed in the leaves. There were 11 *bHLH* genes that showed transcriptional profile differences between artificially inoculated powdery mildew and naturally infected powdery mildew. Among these, three *bHLH* genes (*bHLH162-1*, *bHLH162-2*, *bHLH87*) showed the highest expression levels in healthy leaves (LH), while five *bHLH* genes (*bHLH162-3*, *bHLH25*, *bHLH92*, *bHLH041-1*, *bHLH041-2*) were most highly expressed in leaves infected with powdery mildew (LPM) (Figure 5A). Since artificial inoculation on copper-colored leaves achieved the highest infection efficiency, the expression patterns of these 11 differentially expressed *bHLH* genes were analyzed across different leaf developmental stages. The results demonstrated that the expression levels of *bHLH87* and *bHLH94* decreased from the copper-colored stage to the mature stage (Figure 5B). Analysis of cis-acting elements in the promoter regions of these 11 *bHLH* genes revealed numerous light-responsive elements (e.g., G-box), hormone-responsive elements (gibberellin (GA), abscisic acid (ABA), SA, MeJA), and transcription factor binding sites (MYB transcription factor (MYB), myelocytomatosis proteins (MYC), ethylene responsive factor (ERF)). These findings collectively suggest that these genes play roles in integrating light, hormonal, and transcriptional signals during defense responses (Figure 5C).

### 2.5. Phylogenetic Relationships Between Plant Disease-Related bHLH Proteins and bHLH Family Members in Rubber Trees

**Figure 6:**
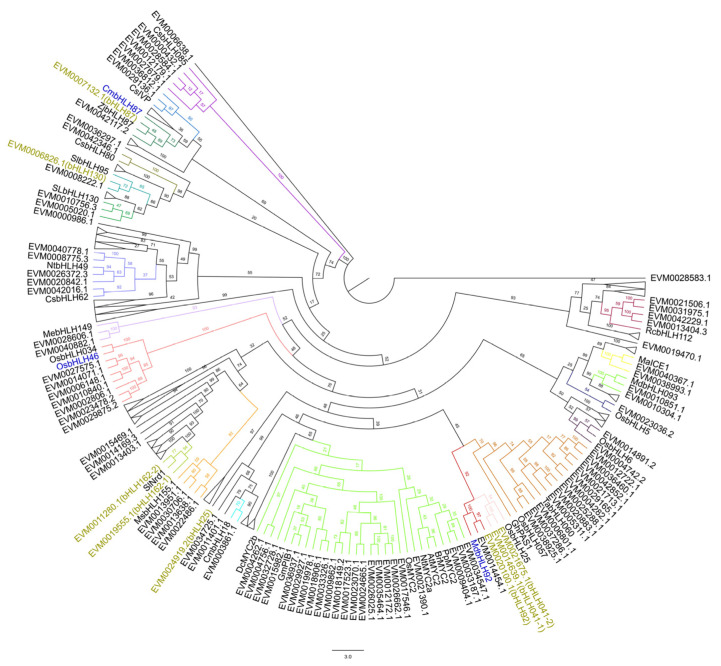
The phylogenetic relationship between other plant disease-related bHLH proteins and the bHLH family members of *Hevea brasiliensis*.

Based on literature reports, 33 *bHLH* genes have been identified as clearly associated with plant disease resistance. An overview of the species to which these genes belong and their functions is provided in Table S2. Among them, two *bHLH* genes are related to plant powdery mildew: *MdbHLH92* (*Malus domestica*) and *CmbHLH87* (*Cucurbita moschata* Duch.). A phylogenetic tree was constructed including these 33 plant bHLH members and 204 bHLH members from rubber trees (Figure 6). The results showed that MdbHLH92 clustered with EVM0020100.1 (bHLH92) and EVM0014454.1, forming one branch, while CmbHLH87 clustered with EVM0042117.2 and EVM0007132.1 (bHLH87), forming another branch. Notably, EVM0020100.1 (bHLH92) and EVM0007132.1 (bHLH87) exhibited significant differential expression in response to powdery mildew treatment. In summary, gene expression patterns, promoter region analysis, and phylogenetic inference suggest that EVM0007132.1 (bHLH87) plays a role in the resistance of rubber trees to powdery mildew.

### 2.6. Expression Patterns of Key bHLH Genes Under Erysiphe quercicola and Hormone Treatments

After inoculation with *Erysiphe quercicola*, the expression level of *HbbHLH87* peaked at 0 h. From 2 h post-treatment onward, it exhibited a significant and continuous decline, remaining at a low level from 24 to 96 h. This downward trend was consistent with previous RNA sequencing analyses. *HbbHLH162-2* was highly expressed from 0 to 2 h post-inoculation, then decreased significantly from 3 h onward, maintaining a low level from 6 to 96 h. The expression level of *HbbHLH25* gradually increased during the early infection stage, peaked at 12 h, and then slowly declined by 24 h. Following treatment with 10 μmol/L MeJA, the expression level of *HbbHLH87* continued to increase, peaking at 24 h, and was significantly higher than at other time points. *HbbHLH162-2* expression increased significantly 2 h post-treatment, peaked at 6 h, and then decreased markedly from 10 to 24 h. *HbbHLH25* showed a rapid response at 0.5 h, with a significant increase in expression. Its expression then decreased during the subsequent period before peaking again at 24 h. Under treatment with 10 μmol/L SA, the expression level of *HbbHLH87* increased over time, peaking at 10 h, but decreased by 24 h. The expression level of *HbbHLH162-2* was significantly higher at 6 h post-treatment compared to other time points, exhibiting an overall increasing-then-decreasing trend. The expression level of *HbbHLH25* was significantly higher from 6 to 10 h post-treatment compared to the initial period (0–0.5 h).

## 3. Discussion

bHLH is present in all eukaryotes and represents one of the largest transcription factor families identified in plants. bHLH proteins regulate the expression of genes involved in plant growth and development through transcriptional activation or repression, as well as genes associated with responses to environmental stimuli, including biotic stresses such as diseases and pests, and abiotic stresses such as high temperature and drought. In this study, a total of 204 bHLH members were identified in the genome of the rubber tree cultivar CATAS73397, all of which were localized to the nucleus. This number exceeds the 148 bHLH members reported in the related species cassava (*Manihot esculenta Crantz*) [15]. Based on the characteristics of the bHLH-related domains, the 204 bHLH proteins were classified into 14 groups, with members within the same group exhibiting similar motif distribution patterns. Notably, most bHLH family members in the AtIND group were not expressed in leaves, whereas most genes in the AtPIF group showed high expression levels in leaves. In Arabidopsis thaliana, members of the AtIND group are primarily involved in seed dispersal, fruit development, and reproductive development [21,22,23]. Members of the AtPIF group, which include phytochrome-interacting factors (PIF1, PIF3, PIF4, PIF5, PIF6, and PIF7), often interact with phytochromes and participate in phytochrome signaling pathways. These factors regulate seed germination, promote axillary bud elongation, induce leaf senescence, and control growth and developmental processes such as flowering. Additionally, they interact with endogenous plant hormones to modulate plant growth and development [24,25,26]. Based on these findings, it can be inferred that the AtPIF members in the rubber tree may play a significant role in the infection of rubber tree leaves by powdery mildew.

The primary mode of replication for the bHLH family members in rubber trees was WGD or segmental duplication. There was strong collinearity among the bHLH members, and they also exhibited collinearity with 99 AtbHLH members from *Arabidopsis thaliana*. Based on the classification of bHLH family members in *Arabidopsis thaliana*, the 204 bHLH proteins in rubber trees were divided into 15 subfamilies [27]. In contrast, the 195 SlbHLH proteins identified in the tomato genome were classified into 27 subfamilies according to Pires’ research [28,29]. Wang et al., referencing Heim’s classification of bHLH subfamilies in *Arabidopsis thaliana*, divided the 180 bHLH family members of rubber trees into 23 subfamilies [30]. The 204 bHLH proteins of rubber trees were categorized into 15 subfamilies based on the functional roles of bHLH family members in *Arabidopsis thaliana*. Some family members were involved in abiotic and biotic stress responses, primarily related to JA response, cold response, iron homeostasis, salinity, and drought stress, while most family members participated in processes related to plant growth and development, such as photosynthesis, flower development, root development, and cell elongation [27].

**Figure 7:**
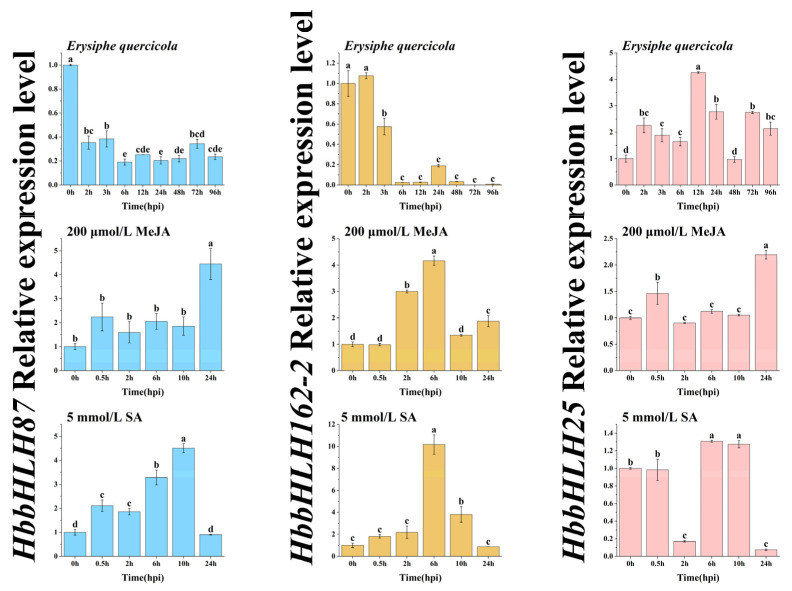
In the leaves of *Hevea brasiliensis*, the expression patterns of *HbbHLH87*, *HbbHLH162-2*, and *HbbHLH25* at different time points after infection by Erysiphe quercicola and treatment with SA and MeJA hormones. The experiments were independently replicated a minimum of three times. The data are presented as the mean ± standard error (n = 3), with distinct letters signifying statistically significant differences (*p* < 0.05) as determined by Duncan’s multiple range test.

There were 11 *bHLH* genes that exhibited differential expression in both natural infection and artificial inoculation with *Erysiphe quercicola*. Among them, the differentially expressed bHLH proteins with a clear systematic evolutionary relationship to known powdery mildew-resistant bHLH proteins (MdbHLH92, CmbHLH87) included EVM0020100.1 (bHLH92) and EVM0007132.1 (bHLH87). Furthermore, ZjbHLH87 and CmbHLH87 showed a close phylogenetic relationship, while MdbHLH92 was closely related to rice OsbHLH25 in terms of evolutionary lineage (Figure 6). MdERF100 mediates resistance to powdery mildew by regulating the JA and SA signaling pathways, whereas MdbHLH92 interacts with MdERF100 and participates in the defense response of *Arabidopsis thaliana* to powdery mildew [31]. Upon pathogen attack, rice plants increase the production of H_2_O_2_. This H_2_O_2_ directly oxidizes bHLH25 at the methionine 256 site within the cell nucleus. By sensing H_2_O_2_, bHLH25 enhances rice resistance to various pathogenic bacteria. Figure 7 shows that under *Erysiphe quercicola* inoculation and 10 μmol/L SA treatment, the expression of *HbbHLH25* was significantly upregulated. This observation aligns closely with the mechanism in rice, where OsbHLH25 enhances disease resistance by sensing H_2_O_2_ [32]. Among pumpkin varieties resistant to powdery mildew, the expression of *CmbHLH87* was significantly suppressed by powdery mildew. Figure 7 illustrates that EVM0007132.1 (*HbbHLH87*) was significantly downregulated after powdery mildew infection (72–96 h post-inoculation), consistent with the inhibition of *CmbHLH87* expression observed in resistant pumpkin varieties. In tobacco plants, ectopic expression of CmbHLH87 alleviates powdery mildew symptoms on leaves, accelerates cell necrosis, and increases H_2_O_2_ accumulation [20]. The SJP39 protein specifically binds to ZjbHLH87. By preventing the degradation of ZjbHLH87, it causes abnormal accumulation of this transcription factor complex in jujubes, further exacerbating the inhibition of key enzymes ZjKAO and the response factor ZjGRP11 involved in gibberellin synthesis. This process contributes to the development of jujube witches’ broom (JWB) disease [33]. Additionally, analysis of cis-acting elements in the promoters revealed that the differentially expressed bHLH proteins contain numerous light-responsive and hormone-responsive elements. Plants regulate growth, stress resistance, and other physiological processes through hormones. Among these, SA and MeJA, as core regulatory factors in plant defense signaling pathways, play crucial roles in resisting biotic stress [34]. The expression level of rubber tree *HbbHLH162-2* was significantly upregulated in response to MeJA and SA stimulation. In apple, MdbHLH162 integrates GA and JA signals to negatively regulate anthocyanin biosynthesis. Rubber tree HbbHLH162-2 may similarly participate in disease resistance by integrating JA and SA signals [35]. Exogenous hormones such as ABA, MeJA, ethephon (ETH), and SA induce the expression of *CmbHLH87* in pumpkin. The expression of rubber tree *HbbHLH87* was significantly upregulated following MeJA and SA treatment, confirming functional conservation with CmbHLH87 in pumpkin. This suggests that rubber tree bHLH proteins may have similar disease resistance functions to CmbHLH87 [20]. The *HbbHLH25* gene was also significantly expressed under MeJA and SA induction, indicating its potential involvement in the H_2_O_2_-mediated disease resistance response through the SA signaling pathway.

## 4. Materials and Methods

### 4.1. Data Downloading and Identification of the bHLH Gene Family in the Whole Genome

The genome assembly and annotation files of the rubber tree cultivar CATAS73397 were downloaded from https://ngdc.cncb.ac.cn/gwh/Assembly/83510/show (accessed on 6 March 2024). Using the GXF sequence extraction tool in TBtools, CDS sequences were extracted and translated into protein sequences [36]. The raw RNA sequencing data of rubber tree (CATAS73397) leaves at four representative stages of leaf development—bronze (SRR3136185), color-change (SRR3136188), pale-green (SRR3136190), and mature (SRR3136192) were downloaded from NCBI [37]. Members of the bHLH family were identified through PlantTFDB from https://planttfdb.gao-lab.org/prediction.php (accessed on 10 March 2024) and BLASTp searches (E-value = 10^−5^), with false positives removed using the Conserved Domain Database (CDD) from https://www.ncbi.nlm.nih.gov/Structure/cdd/wrpsb.cgi (accessed on 16 March 2024). The physicochemical properties of the rubber tree bHLH family members were predicted and analyzed using ProtParam form https://web.expasy.org/protparam (accessed on 14 March 2024). Subcellular localization predictions for these bHLH proteins were performed using the DeepLoc-2.1 tool from https://services.healthtech.dtu.dk/services/DeepLoc-2.1 (accessed on 16 March 2024) [38].

### 4.2. bHLH Gene Structure, MEME Clustering Analysis and Collinearity Analysis

Motif analysis of the bHLH family members in rubber trees was performed using the online platform MEME from https://web.mit.edu/meme/current/share/doc/meme.html (accessed on 16 March 2024). Domain analysis of the bHLH family members was conducted using the Conserved Domain Database online platform. Images depicting MEME clusters, gene structures, and conserved domains were generated using the Gene Structure View tool in TBtools [36]. Collinearity among the bHLH family members in rubber trees was analyzed with MCScanX software, which also statistically determined the types of gene duplications present [39]. Additionally, collinearity analysis between rubber tree and *Arabidopsis thaliana* bHLH family members was performed using MCScanX [39]. The chromosomal positions of bHLH genes were identified based on the GFF annotation file of the rubber tree genome [36].

### 4.3. Construction of the bHLH System Evolutionary Tree

Using MUSCLE, a multiple sequence alignment was performed for 204 members of the rubber tree bHLH family and 172 members of the *Arabidopsis thaliana* bHLH family [40]. IQ-TREE employed the maximum likelihood method (Model: JTT + G4) to construct the ML tree [41]. Functions of bHLH members related to plant diseases were identified through keyword searches in the PubMed database and subsequently summarized. Based on genetic characteristics reported in the literature, protein sequences were downloaded from NCBI and organized. Thirty-three bHLH members with well-characterized functions in other plants were combined with the 204 rubber tree bHLH members to construct an ML tree. FigTree v1.4.4 was used to visualize and enhance the phylogenetic tree of the bHLH family members.

### 4.4. Analysis of Cis-Acting Elements in the Promoter Regions of bHLH Family Members

The promoter region sequence (2000 bp upstream of the coding sequence) of the target gene was extracted using the genome assembly and annotation files. These promoter sequences were analyzed and predicted using the PlantCARE database, and unknown cis-regulatory elements were identified and cataloged. Based on the PlantCARE prediction results, graphical representations were generated using the Simple BioSequence Viewer tool within the TBtools software [36].

### 4.5. RNA Sequencing Analysis

In Danzhou City, Hainan Province, six samples each of infected and healthy leaves were collected from three rubber trees cultivar CATAS73397 and immediately frozen in liquid nitrogen. RNA was extracted from the leaves, and RNA sequencing libraries were constructed. Sequencing libraries were generated using NEBNext UltraTM RNA Library Prep Kit for Illumina (NEB, Ipswich, MA, USA). Raw data were sequenced using the Illumina platform. The Raw data had been uploaded to the China National Center for Bioinformation (PRJCA045342). Sequencing quality was assessed using FastQC and Trimmomatic, and the raw RNA sequencing data were filtered to obtain clean reads. RNA sequencing sequences were aligned, assembled, and quantitatively analyzed using HISAT2 and StringTie software [42,43]. The reference genome was used in the transcriptome analysis of this study was the genome of the rubber tree (CATAS73397) [44]. Differential expression analysis was performed using DESeq2. Significantly differentially expressed genes (DEGs) were identified using the criteria |log_2_Fold Change| ≥ 1 (equivalent to Fold Change ≥ 2) and an adjusted *p*-value (FDR) < 0.01 [45]. A heatmap was generated based on the FPKM values of genes in the RNA sequencing. The spore suspension spraying inoculation method was used to artificially inoculate *Erysiphe quercicola* onto the leaves of the rubber tree cultivar CATAS73397 [10]. Samples were collected at 0, 8, and 36 h post-infection with *Erysiphe quercicola*, with synchronous controls established (data not yet published).

### 4.6. Relative Fluorescence Quantitative Analysis

Selected healthy leaves from CATAS73397 rubber trees, grown under consistent conditions, were sprayed with a suspension of *Erysiphe quercicola* spores at a concentration of 1 × 10^5^ spores/mL. Hormone treatments were also applied by spraying 5 mmol/L SA and 200 μmol/L MeJA. The solvent for the hormones was 0.05% (*v*/*v*) Tween 20 surfactant. The control group was treated with 0.05% (*v*/*v*) Tween 20. All treated leaf samples were collected at various time points, immediately frozen in liquid nitrogen, and stored at −80 °C for future use. Total RNA was extracted from all samples using the TianGen Plant Polysaccharide Multi-Step Extraction Kit (TianGen Bio-Technology, Beijing, China). One microgram of total RNA was reverse-transcribed using the RevertAid RT Kit (Thermo Scientific, Waltham, MA, USA) according to the manufacturer’s instructions. Expression pattern analysis following *Erysiphe quercicola* inoculation and hormone treatments was performed using ChamQ SYBR Color qPCR Master Mix (Nanjing Vazyme Biotechnology Co., Ltd., Nanjing, China) on a CFX96 Touch System (Bio-Rad, Hercules, CA, USA), with the *HbActin* gene serving as the internal reference. The primer information used in this study was presented in Table S3. The 20 μL reaction mixture consisted of 10 μL ChamQ SYBR Color qPCR Master Mix, 0.4 μL forward primer, 0.4 μL reverse primer, 1 μL cDNA template, and 8.2 μL ddH2O. The thermal cycling conditions were as follows: initial denaturation at 95 °C for 10 s; 40 cycles of denaturation at 95 °C for 10 s and annealing at 60 °C for 30 s; followed by melting curve analysis at 95 °C for 15 s, 60 °C for 1 min, and 95 °C for 15 s. Relative expression levels were calculated using the 2^−ΔΔCt^ method. The data are presented as the mean ± standard error (n = 3), with distinct letters signifying statistically significant differences (*p* < 0.05) as determined by Duncan’s multiple range test.

## 5. Conclusions

In the rubber tree cultivar CATAS73397, 204 members of the basic helix–loop–helix (bHLH) transcription factor family were systematically identified. The structural characteristics and evolutionary patterns of these genes were analyzed, and genes related to resistance to powdery mildew were identified. WGD was identified as the primary mode of family expansion. 11 *bHLH* genes responded to both natural infection and artificial inoculation with *Erysiphe quercicola*. Phylogenetic analysis, integrating functions of bHLH members related to disease resistance in other plants, identified eight rubber tree bHLHs closely related to powdery mildew resistance-associated bHLHs from other species. *HbbHLH87*, *HbbHLH162-2*, and *HbbHLH25* were all regulated by *Erysiphe quercicola*, SA, and MeJA. This suggests that they may participate in the defense response against powdery mildew through coordinated hormone signal transduction.

## Data Availability

The RNA-seq data generated in this study have been deposited in the China National Center for Bioinformation (CNCB), under accession number PRJCA045342. The data are publicly accessible as of the release date 2025-08-28 via the following URL: https://www.cncb.ac.cn (accessed on 28 August 2025).

## Supplementary Materials

The following supporting information can be downloaded at: https://www.mdpi.com/article/10.3390/plants14213244/s1, Supplement Table S1. the physical and chemical properties as well as subcellular localization predictions of members of the bHLH family; Supplement Table S2. the bHLH transcription factors related to plant disease resistance; Supplement Table S3 RT-qPCR primers information.
